# Unraveling the Role of Dopaminergic and Calretinin Interneurons in the Olfactory Bulb

**DOI:** 10.3389/fncir.2021.718221

**Published:** 2021-10-08

**Authors:** Simona Capsoni, Alex Fogli Iseppe, Fabio Casciano, Angela Pignatelli

**Affiliations:** ^1^Department of Neuroscience and Rehabilitation, University of Ferrara, Ferrara, Italy; ^2^Bio@SNS Laboratory of Biology, Scuola Normale Superiore, Pisa, Italy; ^3^Department of Translational Medicine and LTTA Centre, University of Ferrara, Ferrara, Italy; ^4^Interdepartmental Research Centre for the Study of Multiple Sclerosis and Inflammatory and Degenerative Diseases of the Nervous System, University of Ferrara, Ferrara, Italy

**Keywords:** interneurons, olfactory bulb, dopaminergic cell, calretinin cell, adult neurogenesis

## Abstract

The perception and discriminating of odors are sensory activities that are an integral part of our daily life. The first brain region where odors are processed is the olfactory bulb (OB). Among the different cell populations that make up this brain area, interneurons play an essential role in this sensory activity. Moreover, probably because of their activity, they represent an exception compared to other parts of the brain, since OB interneurons are continuously generated in the postnatal and adult period. In this review, we will focus on periglomerular (PG) cells which are a class of interneurons found in the glomerular layer of the OB. These interneurons can be classified into distinct subtypes based on their neurochemical nature, based on the neurotransmitter and calcium-binding proteins expressed by these cells. Dopaminergic (DA) periglomerular cells and calretinin (CR) cells are among the newly generated interneurons and play an important role in the physiology of OB. In the OB, DA cells are involved in the processing of odors and the adaptation of the bulbar network to external conditions. The main role of DA cells in OB appears to be the inhibition of glutamate release from olfactory sensory fibers. Calretinin cells are probably the best morphologically characterized interneurons among PG cells in OB, but little is known about their function except for their inhibitory effect on noisy random excitatory signals arriving at the main neurons. In this review, we will mainly describe the electrophysiological properties related to the excitability profiles of DA and CR cells, with a particular view on the differences that characterize DA mature interneurons from cells in different stages of adult neurogenesis.

## Introduction

The sense of smell, or olfaction, is one of the oldest senses (Sarafoleanu et al., 2009). In humans, it is believed that in everyday life the sense of smell is less important than hearing or vision. This statement was based on the small size of the olfactory bulb compared to the whole brain and that initially, despite the fact that 1,500 genes encoding for olfactory receptors are found in our genome, thus accounting for the largest part of it (Young and Trask, 2002), only 390 genes were found to be functional and the remaining were considered pseudogenes (Glusman et al., 2001). However, contrary to what it was commonly believed, the human olfactory bulb is in reality quite large in absolute terms and contains a comparable number of neurons to other mammals (Bushdid et al., 2014; McGann, 2017). In addition, most of the pseudogenes were subsequently found to be functional receptors (Olender et al., 2016; Prieto-Godino et al., 2016). For this reason, humans can distinguish at least 1 trillion of different odors (Bushdid et al., 2014).

In mammals, the olfactory system is capable of odor detection, odor discrimination, and olfactory memory and employs these capabilities in a wide range of behaviors. Indeed, olfaction is essential: (1) to find food and determine the preference for it, (2) in regulating appetite, (3) when exploring the surrounding environment, (4) for social interactions and during behaviors related to courtship and mating; (5) in empathic behaviors (Spinella, 2002; Sarafoleanu et al., 2009; Fine and Riera, 2019). In humans, it plays an important site of integration among different senses such as in the perception of taste (Rolls, 2019) and during eyesight (Gottfried and Dolan, 2003). Moreover, it contributes to the central regulation of gastrointestinal functions (Kitamura et al., 2010) and, thanks to the connections between the olfactory and limbic systems, it is involved in psychosocial interactions (Li and Liberles, 2015; Cherry and Baum, 2020), aggressive behaviors (Ferris et al., 2008), learning and the memory of fear (Mouly and Sullivan, 2010).

Thus, the sense of smell strongly impacts everyday life. Indeed, olfactory dysfunctions significantly worsen physical well-being, quality of life, everyday security, and are associated with increased mortality in older adults and it is among the prodromic symptoms of several neurodegenerative diseases (Attems et al., 2015).

A complex circuitry is the basis of the correct functioning of the sense of smell. In this review, we will focus on the description of two populations of olfactory bulb interneurons, the dopaminergic and calretininergic cells that are important actors in odor discrimination.

## Neuronal Circuit of the Olfactory System

Odors are detected and recognized by odorant receptors expressed on olfactory sensory neurons (OSNs) located in the olfactory epithelium. After binding to the specific receptor, the information is then transduced in an electrical signal that later reaches the olfactory bulb (OB) where the first processing of sensory signals takes place. Indeed, the transmission of sensorial information is not simply conducted by the projection neurons, but the signals are revised and modulated by the local circuitry mainly by the action of interneurons. Then, through projection neurons, the signal reaches higher-order areas of the brain such as the superficial plexiform layer of the accessory olfactory nucleus, the piriform cortex in the temporal lobe, periamygdaloid, and lateral entorhinal cortices, taenia tecta, the anterior hippocampal continuation, indusium griseum, and the olfactory tubercle (Shipley and Ennis, 1996). Additional areas where efferent fibers from the OB can be found are the amygdala (Kang et al., 2011) and the hypothalamus (Scott and Pfaffmann, 1967; Price, 1985; Hatton and Yang, 1989).

In this review, we will focus on the role of the piriform cortex (PCx) in odor processing. The PCx is a three-layer paleocortical structurally different from other neocortical sensory areas and more similar to hippocampal, cerebellar and prefrontal cortical areas (Neville and Haberly, 2004). In the PCx, contrary to the OB, neurons respond to multiple odors (Stettler and Axel, 2009), and thus PCx function does not depend on a spatial order (Miura et al., 2012). Consequently, PCx integrates input coming from different glomeruli. However, PCx neurons can support odor identification, odor discrimination learning and odor value for a complete discussion see (Bekkers and Suzuki, 2013; Blazing and Franks, 2020).

Concerning PCx circuits, it has been found that the piriform cortex can also project directly to prefrontal, amygdaloid, entorhinal, and perirhinal cortex where it can play an associative function, implying that the OB can function similarly to other primary areas of other sensory systems (Johnson et al., 2000; Bekkers and Suzuki, 2013). Here we will briefly focus on the connection between the PCx and the thalamus.

In general, sensory systems use the thalamus as an intermediate step to reach primary cortical areas where other sensory information is processed. In the past it has believed that olfaction is independent from the thalamus but it is now accepted that the odorant information is first processed by the PCx which then projects to the mediodorsal nucleus of the thalamus (Price and Slotnick, 1983; Bay and Cavdar, 2013), giving rise, with the reciprocal connection with the orbitofrontal cortex, to the olfactory trans-thalamic pathway (Krettek and Price, 1977). The role of the mediodorsal thalamus in odor elaboration has not been fully clarified but it is supposed to contribute to odor perception and learning discrimination. A detailed description is beyond the scope of this review and readers can find more information in Courtiol and Wilson (2015).

**Figure 1:**
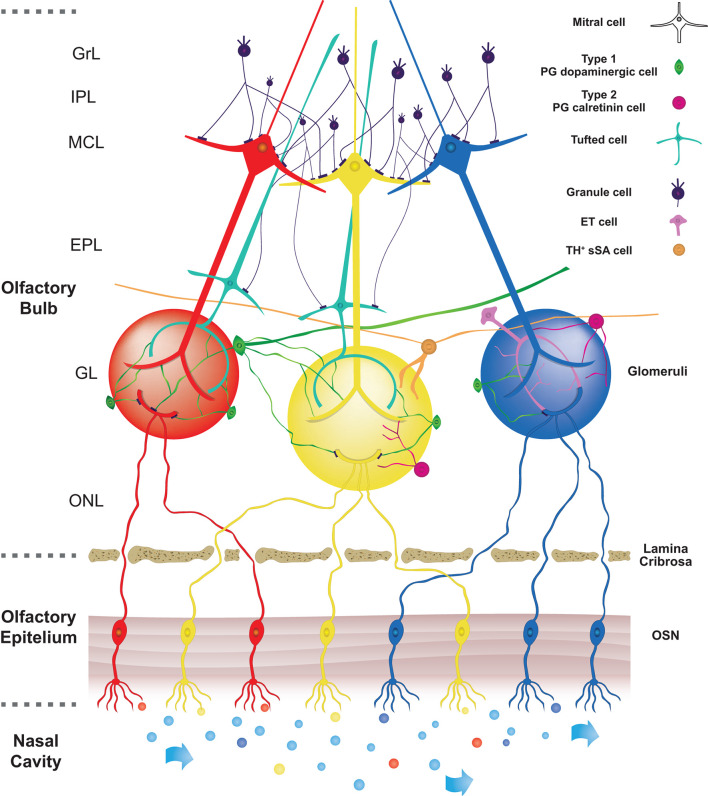
Schematic pathway of the main neuronal connections in OB. In the nasal cavity, airflow transports different odorants (represented by different colours) to specific olfactory receptors expressed on olfactory sensory neurons (OSNs) localized in the olfactory epithelium (OE). Olfactory information is then transduced in an electrical signal to later reach the olfactory bulb (OB). Olfactory sensory neurons send their axons through the lamina cribrosa to reach the core of corresponding glomeruli, which consists of spherical structures located in the olfactory bulb. Axons of OSNs, expressing the same odorant receptors, are projected into the same glomeruli, so each glomerulus is linked to a single odorant receptor. Within the glomerular core, OSNs axons make synapses with apical dendrites of projections corresponding to OB neurons (Mitral and Tufted cells). On the surface of glomeruli are located periglomerular cells (PG). These cells project their dendrite into the glomerulus and make reciprocal synapses with projections neurons and some of them with axons of OSNs. Based on their synaptic connections with axons of OSNs, two subtypes of PG cells are described. Type 1 PG cells, belonging to dopaminergic PG cells, are synaptically connected both with axons of OSNs and dendrites of other neurons in the olfactory bulb. On the other hand, type 2 PG cells, belonging to calretinin PG cells, are not synaptically connected with axons of OSNs. The figure shows the three subtypes of DA cells present in the glomerulus area according to Nagayama et al. (2014) and Kosaka et al. (2020). The PG-DAs are represented in green. The large ones that have an axon and dendrites that enter more glomeruli and the small ones that in most cases enter one or a few glomeruli and do not have an axon have been represented. The ET cells and those belonging to the subtype of TH^+^ sSA were represented too.

In this review, we will focus in more detail on the circuitry within the olfactory bulb. In Figure 1 we illustrate a very schematic pathway of the main connections that concern the transmission of the olfactory signal. The anatomical structure of OB can be described as a sequence of five, concentric layers of cells and fibers. The first one is the olfactory nerve layer, located on the surface of the OB, and it is formed by the afferent projections from the olfactory epithelium. The terminals of these axons enter the second, glomerular layer (GL) which consists of spherical structures called glomeruli from which it takes the name. Within this layer, the axons of OSNs constitute the core of the glomerulus and create synapses with the apical dendrites of projecting neurons (mitral and tufted cells) and at the same time with interneurons surrounding the glomeruli which are called juxtaglomerular cells (JGcs). In a truly surprising way, it has been demonstrated the presence of a very precise mapping for which OSNs expressing the same odorant receptors project and converge their axons into the same glomeruli (Mori and Sakano, 2011).

The third layer of the OB is the external plexiform layer (EPL), composed of the cell body of tufted cells, the basal dendrites of tufted and mitral cells and the apical dendrites of the principal type of interneurons, the granule cells (Gcs). The cell bodies of mitral cells are found in the fourth layer (mitral layer, MCL) which takes the name from those types of cells.

Mitral and tufted cells are two recognized types of projection neurons. Both types of cells send their axons to the olfactory cortex. However, before exiting the OB, modulation of the signal collected by the mitral and tufted projection neurons occurs in the external plexiform layer where their secondary dendrites are elongated and form reciprocal synapses with the dendrites of the granule cells.

The fifth layer contains axons from mitral, collateral axons from external tufted cells and dendrites of granule cells. It is called internal plexiform layer (IPL).

The sixth layer is formed by the somata of granule cells (granular layer, GrL). The morphology and physiology of granule cells have been thoroughly described in previous reviews (Shepherd et al., 2007; Burton, 2017; Takahashi et al., 2018) and thus we will focus on two types of interneurons expressing dopamine and calretinin found in the glomerular layer.

## Juxtaglomerular Interneurons

In the glomerular layer, the spherical glomeruli are enriched with axons and dendritic processes derived from OSN and cells innervating them. Indeed, glomeruli are surrounded by three main types of interneurons. These cells are overall classified as juxtaglomerular cells but, thanks to their different morphology, they can be divided into three distinct subpopulations which are characterized by the absence of projections outside the olfactory bulb: external tufted (ET) cells, short axon surface (sSA) cells and periglomerular cells (Nagayama et al., 2014).

ET cells can be classified into two subtypes according to their morphology. The first subpopulation is represented by ET without secondary dendrites is the less frequent of the two, while ET neurons with secondary dendrites are generally observed close to the border between the glomerular and the external plexiform layer (Nagayama et al., 2014).

sSA neurons are characterized by long, interglomerular axons connecting a variable number of glomeruli (Kiyokage et al., 2010; Liu et al., 2016) and for this reason, they are believed to be the most frequent origin of interglomerular projections in the OB (Aungst et al., 2003; Kiyokage et al., 2010). These cells express tyrosine hydroxylase (TH) and, due to their long interglomerular projections, they are believed to coincide with the axonic dopaminergic neurons described by Galliano and Korshunov at the border between the GL and the EPL layers (Galliano et al., 2018; Korshunov et al., 2020b).

Since dopaminergic neurons are believed to be the most important cells providing interglomerular connections (Aungst et al., 2003; Kosaka and Kosaka, 2008a; Kiyokage et al., 2010), sSA neurons were believed to be the only type of neuron expressing dopamine. However, a population of DA interneurons characterized by the absence of axons (Chand et al., 2015; Galliano et al., 2018) has been classified, based on the morphology of their dendritic arborization, as PG cells (Kosaka and Kosaka, 2009a, 2011, 2016; Korshunov et al., 2020a; Kosaka et al., 2020).

Thus, in the next chapters of this review, we will focus on the description of the properties characterizing PG cells, since they represent the most numerous types of neurons. Indeed, it has been calculated that they are almost the 60% of the total interneurons of the OB (Parrish-Aungst et al., 2007). Another characteristic of these neurons is related to the diameter of the cell body which was found to be in the range of 6–8 μm, thus making these cells the smallest among the different JGcs (Pinching and Powell, 1971a; Nagayama et al., 2014). In addition, in most cases, the dendrites departing from these neurons innervate only one glomerulus and less frequently more than one (Pinching and Powell, 1971b).

From this anatomical observation, PG interneurons surrounding the glomerulus are classified into two cell subtypes, type 1 and type 2, based on the nature of synaptic interaction they make with the axon terminals of the olfactory sensory neurons. Indeed, while both types of PG cells extend their dendritic processes in regions of the glomerulus devoid of olfactory nerves, only type 1 PG interneurons make synapses with olfactory nerves in ON zones (Kosaka et al., 1998). This observation was confirmed by electrophysiological studies performed in OB slices in which delays in response to the electric stimulation of olfactory nerve bundles in the olfactory nerve layer have been observed (Shao et al., 2009; Kiyokage et al., 2010).

Another characteristic that helps in classifying PG interneurons is their neurochemical nature, associated with the expression of neurotransmitters or calcium-binding proteins. PG cells can be divided into three main subtypes based on the immunoreactivity to tyrosine hydroxylase, calretinin, calbindin-28K (CB) (Kosaka et al., 1998; Kosaka and Kosaka, 2011).

Since the immunoreactivity for these compounds is largely mutually exclusive, these three cellular subtypes are virtually non-overlapping (Kosaka and Kosaka, 2007; Parrish-Aungst et al., 2007). The functional roles of each PG cells subtype in odor processing are still being the object of investigation. However, it has been reported that all PG interneurons synthesize γ-aminobutyric acid (GABA) and express at least one of the two glutamic acid decarboxylase (GAD) isoforms, GAD65 and GAD67, except for a small percentage of CR-positive neurons (Kosaka, 2007). Therefore, they can be classified as inhibitory neurons. In more than half of the JG cells population, one or both isoforms are expressed (Parrish-Aungst et al., 2007; Whitman and Greer, 2007).

Several studies have also tried to calculate how many PG cells belong to the different groups. Results suggest that CR cells are the most abundant subtype, while the CB-positive cells are the fewest, but these proportions vary among the different studies, probably depending on the method employed in the analysis (Kosaka and Kosaka, 2007; Panzanelli et al., 2007; Parrish-Aungst et al., 2007; Whitman and Greer, 2007).

The different subtypes of interneurons present in the glomerulus also have different temporal patterns of neurogenesis throughout life. PG cells, together with granular cells, represent a neuronal population that is produced both during embryonic life and in adulthood (Altman, 1962, 1969; Hinds, 1968). PG cells expressing TH and CB are added mainly at embryonic days 12.5–15.5, while interneurons expressing CR increase in number during adulthood (Batista-Brito et al., 2008; Nagayama et al., 2014; Kim et al., 2020).

## DA Cells in the Olfactory Bulb

Juxtaglomerular dopaminergic neurons of the olfactory bulb represent the most numerous endogenous dopamine-producing cells in the forebrain (Guyenet and Crane, 1981; Cave and Baker, 2009) and they are identified in the A16 group of neurons in the standard classification (Bjorklund and Dunnett, 2007).

Dopaminergic neurons are purposely located at the entrance of the bulbar circuitry, in this way they are directly in contact with the olfactory nerve terminals and sustain odor processing and adaptation processes of the bulbar network to external environments.

Mature DA neurons in OB have been almost exclusively described in the glomerular layer (Halasz et al., 1981), and there is consensus on the fact that they co-release dopamine and GABA from separate pools of vesicles (Maher and Westbrook, 2008; Borisovska et al., 2013).

Thanks to the use of TH as a marker of dopaminergic neurons, it has been estimated that 10–16% of the neurons in the glomerular layer of adult animals are indeed DA (McLean and Shipley, 1988; Panzanelli et al., 2007).

In the last decades, there has not been a unison agreement on the classification based on the morphology and nomenclature of the DA cells present in the glomerular layer. One classification distinguishes three types of cells: (1) PG-DA (Kosaka et al., 1985; Gall et al., 1987); (2) a subpopulation of external tufted cells which, differently from the ET neurons present in the EPL, are not projection neurons (Gall et al., 1987; Halász, 1990; Kosaka and Kosaka, 2011) and (3) a subpopulation of short axon cells (SA) (Kiyokage et al., 2010; Liu et al., 2013; Nagayama et al., 2014; Sanz Diez et al., 2019) with soma in the glomerular layer that has been called superficial (sSA) and are distinguished from those present in the granule cell layer which are called deep SA (dSA). Both subtypes are important for intrabulbar connections. Several subtypes of dSA cells have been reported to play the role of mediating GABAergic inhibition on interneurons PG and Gr (Eyre et al., 2008; Boyd et al., 2012; Burton, 2017). The superficial SA cells instead release both GABA and DA in other glomeruli and produce a temporally biphasic inhibition-excitation response in external tufted cells (Liu et al., 2013; Whitesell et al., 2013; Sanz Diez et al., 2019). A detailed analysis using the retrograde tracing technique showed that these cells possess long neuronal processes and are positive not only for the TH marker but also for the GAD67 isoform (Kiyokage et al., 2010).

The sSA TH^+^/GAD67^+^ cells differ from the classic sSA cell reported by Pinching and Powell previously (Pinching and Powell, 1971a,b) because they have an axon that extends for ~1 mm, and its dendrites make contacts with up to 50 glomeruli, while sSA cell has classically been described to have a shorter axon extending only for one to two glomeruli while dendrites avoid glomeruli (Nagayama et al., 2014).

In the second type of classification, it appears that DA neurons present in the glomerulus layer fall into at least two subpopulations that are differentiated by a bimodal distribution of the size and diameter of the soma (Pignatelli et al., 2005; Kosaka et al., 2020) and last for the presence or absence of an axon (Chand et al., 2015; Galliano et al., 2018). Larger neurons probably might correspond to external tufted cells or sSAc (Kosaka et al., 2020) and the smallest to PG cells (Halasz et al., 1981; Davis and Macrides, 1983; Hoogland and Huisman, 1999; Pignatelli et al., 2005; Kosaka, 2007; Kosaka and Kosaka, 2008b; Liberia et al., 2012; Pignatelli and Belluzzi, 2017; Kosaka et al., 2020). External tufted neurons were initially thought to be excitatory neurons, and the small PG inhibitory neurons (Halasz et al., 1981; Davis and Macrides, 1983). However, subsequent studies have indicated that both large and small dopaminergic subgroups are GABAergic interneurons (Kosaka et al., 1985, 1987a; Gall et al., 1987).

Experimental analyses confirmed the classification of these two main subtypes in mice, as reported by Kosaka's laboratory. In a first paper, these authors showed that small DA neurons have an average diameter between 8.76 ± 1.58 μm and 10.69 ± 2.70 μm for the large one. In more recent publications the peak and half-width of the diameters measured of the smaller group were 9.9 ± 2.5 μm, and those of the larger group 13.8 ± 2.1 μm (Kosaka and Kosaka, 2008b; Kosaka et al., 2020). Measures performed in our lab confirmed these data, showing values of 5.67 ± 0.96 and 9.48 ± 2.39 μm respectively for small and large DA-PG neurons (Pignatelli et al., 2005). The existence of large and small PG neurons has been confirmed using electrophysiological analysis observing the difference of action potentials that originate in the soma and then propagate to the dendrites or action potentials that originate in the initial segment of the axon and propagate backward into the soma. In our electrophysiological analyses on the excitability of DA-PG cells (Pignatelli et al., 2005) we did not find significant differences between large and small subtypes, but more recent studies have shown greater excitability of the larger cells which could be correlated to differences in excitability parameters such as a lower threshold and rheobase current, faster action potentials rise phase, higher firing frequency and other electrophysiological peculiarities (Chand et al., 2015).

The presence of axons departing from PG interneurons has been for a long time a matter of debate. The analysis of the two cell types by Kosaka's group showed that the larger cell type of PG neurons projects axonal processes toward glomeruli distant from the glomerulus where the soma is located, becoming a neuron that plays a role in interglomerular association (Kosaka and Kosaka, 2008a, 2011, 2016). These data were confirmed using molecular markers of an initial axon segment (Chand et al., 2015), being this parameter useful for the discrimination of large and small DA neurons.

Axon segment markers are absent in most small DA neurons, as shown both *in vivo* (Kosaka and Kosaka, 2011, 2016) and *in vitro* neurons (Chand et al., 2015; Galliano and Grubb, 2016). However, in a recent review, Kosaka et al. reported the presence of rare small DA neurons characterized by thin axon-like processes positive for axon segment markers (Kosaka et al., 2020). Thus, it can be suggested that, although the proposed classification in large and small DA PG interneurons is one mostly used nowadays, large and short axon DA PG neurons might correspond to the short axon cells described in Nagayama et al. (2014).

ET cells (Halasz et al., 1981; Davis and Macrides, 1983), later recognized as GABAergic are glomerular interneurons that can coordinate long-range intrabulbar connections (Kosaka and Kosaka, 2007; Panzanelli et al., 2007; Parrish-Aungst et al., 2007). On one hand, large DA-PG neurons make synapses with granule cells on the opposite edge of the OB, establishing an association inhibitory circuit through which isofunctional odor columns are connected (Schoenfeld et al., 1985; Lodovichi et al., 2003; Kosaka and Kosaka, 2011). Another circuit is characterized by large type DA glomerular neurons which connect ipsilateral glomeruli (Kosaka and Kosaka, 2011).

Concerning small PG neurons, they represent about 85% of DA neurons in the OB (Pignatelli et al., 2005) and they were found to connect to one glomerulus or few glomeruli in their proximity (Kosaka and Kosaka, 2011, 2016; Kosaka et al., 2020).

To conclude, a recent work from the Kosaka group has further improved the classification of previously described bulbar DA cells by integrating them with three other DA cell subtypes (Kosaka et al., 2020), besides large and small DA-PG neurons, bringing to five subpopulations of DA-positive juxtaglomerular neurons. The first group is referred to as the transglomerular neurons which have a medium-sized cell body and dendritic processes that can reach up to 2–6 glomeruli. They are not only expressing TH but also secretagogin. The second group of juxtaglomerular TH neurons has been defined by Kosaka et al. as “incrusting” cell group which is characterized by dendritic branches running through the periphery of one or more glomeruli, decorating them (Kosaka et al., 2020). The last group of neurons include cells that have no axonal processes and are TH and secretagogin positive and are but that, from the morphological point of view, cannot be classified into the previously described groups. In their study, Kosaka et al. suggest naming them oligoglomerular neurons since they extend their dendrites in more glomeruli (Kosaka et al., 2020).

## Physiological Role of Dopaminergic Neurons

Dopaminergic cells play a dual role in controlling the local gain of transmitter release from the terminals of olfactory sensory neurons (Hsia et al., 1999; Ennis et al., 2001; McGann, 2013) and determine inhibition of lateral glomerular output (Liu et al., 2013). In this way, they can influence the basic characteristics of olfactory sensory behavior (Wei et al., 2006; Serguera et al., 2008). Those cells are also responsible for remarkable activity-dependent plasticity, controlling the release of TH and the synthesis of GAD67 in an activity-dependent manner (Baker et al., 1983; Cave et al., 2010; Parrish-Aungst et al., 2011; Banerjee et al., 2013).

In recent years, DA interneurons in the olfactory bulb have been studied because these cells have three interesting characteristics: first, they are extremely plastic (Baker et al., 1983; Bastien-Dionne et al., 2010), second, they are cells anatomically found at the entrance of the bulbar circuit, suggesting an important role in the odor processing process (Borisovska et al., 2013) and, finally, they are cells constantly generated throughout life (Altman, 1969; Betarbet et al., 1996; Baker et al., 2001; Winner et al., 2002; Mizrahi et al., 2006; Ventura and Goldman, 2007; Lazarini et al., 2014). The fact that they can be produced and replaced during the whole life makes them interesting for regenerative medicine studies as a source of new dopaminergic autologous cells to repair brain-damaged areas, such as in Parkinson's disease (Cave et al., 2014).

From the physiological point of view, the importance of DA interneurons in olfaction has been demonstrated in mice in which the knocking out of dopamine receptors or transporters leads to decreased ability to discriminate odors (Wilson and Sullivan, 1995; Tillerson et al., 2006; Taylor et al., 2009). A similar result was obtained when mice with genetically induced mitochondrial dysfunction were analyzed and found that the odor discrimination deficit was paralleled by a decrease in the number of small PG-DA neurons (Paß et al., 2020). Evidence of their role in humans comes from several studies in which it is shown that olfaction impairment in Parkinson's disease is one of the first signs of neurodegeneration, even prodromic to motor deficits (Doty, 2012).

DA-PG neurons are endowed with autorhythmicity which implies a tonic release of neurotransmitter in the synaptic cleft with ON the terminal. The role of this neurotransmitter in the bulbar circuits, as described in the literature, lends itself to an uncertain interpretation, also due to the presence of various dopamine receptors and their different distribution in the OB. The expression of these receptors within the OB has been analyzed using different techniques spanning from *in situ* hybridization, autoradiography, immunohistochemistry combined with electron microscopy. A first study by Meador-Woodruff detected D1 receptor mRNA in the olfactory bulb (Meador-Woodruff et al., 1991) which was then specifically identified to be the subtype D1A while D1B receptors were undetectable (Coronas et al., 1997). In the same study, *in situ* hybridization showed that D1A is localized in the glomerular and granular layers of the OB, while autoradiography using the [^l25^I]SCH23982 ligand demonstrated the presence of D1A not only in the neuropil of the glomerular layer but also in EPL, mitral layer and IPL. D2 receptors were studied more extensively. They were found to be expressed in the neuropil of all olfactory glomeruli and specifically they were identified by electron microscopy on olfactory axons, dendrites of mitral and tufted neurons (Gutierrez-Mecinas et al., 2005). The presence of D2 receptors in the glomerular layer was also confirmed by autoradiography using the [H^3^]quinpirole D2 agonist as a ligand (Levant et al., 1993). Interestingly, D2 receptors were also found to be expressed on type 1, GABAergic PG neurons (Gutierrez-Mecinas et al., 2005), the function of which remains to be discovered. Very few data are available on other DA receptor types. Faint radiolabelled D3 receptor was found in the olfactory nerve layer and the glomerular layer consistently with mRNA distribution (Sokoloff et al., 1990; Bouthenet et al., 1991; Levant et al., 1993; Coronas et al., 1997). D4 receptor mRNA was not found in the OB while D5 receptor was found in the neuropil of the glomerular, IPL, EPL and granular layers (Coronas et al., 1997; Ciliax et al., 2000).

However, despite this heterogeneity, it has been identified that an important functional role is played by D2 receptors. Indeed, DA can exert an inhibitory effect on olfactory sensory fibers after binding to D2 presynaptic receptors and the reduction of calcium influx via N-type calcium channels. This data is confirmed by the fact that antagonizing D2 receptor activity there is an almost 30% increase in levels of intracellular Ca^2+^ (Wachowiak and Cohen, 1999).

## Electrophysiological Characteristics of the da Cells of the Olfactory Bulb

Autorhythmicity is a unique property common to many DA neurons in the Central Nervous System which allows generating rhythmic action potentials even in the absence of synaptic inputs (Grace and Onn, 1989; Hainsworth et al., 1991; Yung et al., 1991; Feigenspan et al., 1998; Neuhoff et al., 2002). The electrophysiological analysis confirmed that also DA cells present in the glomerular layer of the mouse olfactory bulb possess this pacemaker activity but it was not detected in dopaminergic cells of the rat olfactory bulb suggesting a possible species difference (Pignatelli et al., 2005; Puopolo et al., 2005; Korshunov et al., 2020b). Moreover, we can only hypothesize why in previous studies in mice the authorithmicity of these cells was not reported. One hypothesis is that in some electrophysiological experiments performed in tissue slices derived from wild type mice or, in any case, not from TH-EGFP mice it has not been possible to identify beyond any reasonable doubt the specific subtype of the cell recorded in glomerular layer of the OB slices. Since it has been estimated that only 10–16% of the neurons in the glomerular layer are DA and therefore the use of an unsafe preparation can lead to the lack of certainty of the registrations in the searched subtype.

To further strengthen this finding, we found that this autorhythmicity has similar characteristics to those recorded in midbrain dopamine neurons that are spontaneously active (Grace and Bunney, 1984) and where tonic firing is likely important for maintaining background dopamine levels.

**Figure 2:**
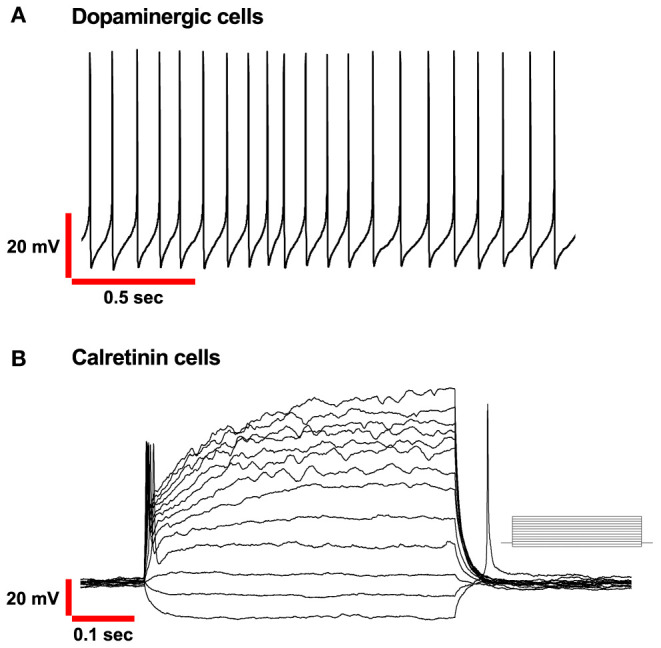
Basic electrophysiological properties of PG Dopaminergic and Calretinin cells in OB. **(A)** Spontaneous autorhythmic action potentials in Dopaminergic cells recorded in current-clamp mode. Frequency range (4–12Hz). **(B**) Voltage responses of Calretinin cells recorded in current-clamp mode. Only a single action potential was elicited to depolarizing injected current (from−15 pA, in increments of 10 pA as shown in the inset).

Autorhythmicity in DA-PG neurons differs from that recorded previously in ET cells in the OB by other researchers (Hayar et al., 2004). DA-PG neurons are spontaneously active and fire in a regular, rhythmic pattern with interspike intervals rather constant in most of the cells (Figure 2A).

In ET cells, on the other hand, the most evident physiological feature is that they spontaneously generate rhythmic *bursts* of action potential that consisted of two or more action potentials on a slow depolarizing envelope.

To describe the ionic basis of rhythm generation in these cells our group used a transgenic mouse expressing an enhanced green fluorescent protein (eGFP) reporter construct under the promoter of TH. This animal model gave us the possibility to identify with absolute precision dopaminergic neurons (TH-GFP cells) in living slices and cultured cells during electrophysiological experiments (Sawamoto et al., 2001).

The complex pattern of voltage-dependent conductance of PG-DA cells that determines their excitability profile, including the pacemaker machinery, has been completely described in our laboratory (Pignatelli et al., 2005, 2012; Pignatelli and Belluzzi, 2017). The first electrophysiological feature of bulbar DA cells is that they have spontaneous activity, mainly within the theta frequency range (4–12 Hz). In all the papers of our group reported here, only cells that showed spontaneous regular firing during electrophysiological recording were taken into consideration.

We used pharmacological approaches and ion substitution methods to describe two large and five small voltage-dependent conductances in these cells.

Following a kinetic characterization, these conductances were analyzed using a Hodgkin-Huxley computational model of DA-PG cells (Pignatelli et al., 2005). We found the two characterized by a large amplitude are a fast transient Na^+^ current and a delayed rectifier current. They generate the action potential in DA-PG neurons.

Two of the small voltage-dependent conductance were identified in a persistent Na^+^ current (INa_P_) and a T-type Ca^2+^ current (ICa_T_) and are responsible for the spontaneous activity of these interneurons (Pignatelli et al., 2012).

The electrophysiological analysis highlighted the presence of two other small conductances, activated by hyperpolarization: an h-current (Fried et al., 2010; Pignatelli et al., 2013) and an inward rectifier current of potassium (KIR) (Borin et al., 2014).

These two currents do not appear to be directly involved in the pacemaking mechanism but play an important role in controlling the excitability of these cells. Both currents are active at rest membrane potential but exert opposite effects on it: h-current leads to depolarization and vice versa the KIR hyperpolarizes it. This control of the membrane potential modulates the excitability of the cell.

Another aspect that should not be underestimated on the role of these currents activated by hyperpolarization is that both are effectively modulated by the mechanisms of the second messenger.

In particular, the two currents h and KIR rely on a cAMP pathway that can be modulated by the several neurotransmitters that are released by the numerous afferents making synapses on DA-PG interneurons.

In the past, it has been shown that afferents from the anterior olfactory nucleus (AON), the piriform cortex and the olfactory tubercle were all involved in odor processing. (Kiselycznyk et al., 2006; Matsutani, 2010; Markopoulos et al., 2012; Rothermel et al., 2014; Otazu et al., 2015; Zhang et al., 2017), although more recently the contribution of olfactory tubercle projections has been challenged by retrograde tracing based on adeno-associated viruses (In 't Zandt et al., 2019). Concerning the inputs to juxtaglomerular interneurons, it has been reported a glutamatergic innervation from the AON to PGCs which leads to their excitation and consequent inhibition of mitral cells (Markopoulos et al., 2012). Juxtaglomerular cells receive also serotoninergic innervation from dorsal and medial raphe nuclei (McLean and Shipley, 1987). Serotonin binds to 5-HT_1A_, 5-HT_2_ and 5-HT_2C_ (Wright et al., 1995). The consequences of this binding have been reported only for 5-HT_2C_ receptors. Indeed, electrophysiological studies showed that the activation of this receptor leads to depolarization of a subset of juxtaglomerular cells (Hardy et al., 2005; Petzold et al., 2009). However, the classification of these cells is unknown.

In the OB the afferents that could be involved with mechanisms regulating pacemaking are the serotonergic ones from the ventral and dorsal raphe nuclei (Araneda et al., 1980), noradrenergic fibers from the locus coeruleus (McLean et al., 1989), cholinergic afferents from the nucleus of the horizontal flap of the diagonal band (Zaborszky et al., 1986) and histaminergic projections from the hypothalamus (Panula et al., 1989).

We demonstrated that the amplitude of the KIR current can be increased when D2 receptors are activated or when DA-PG interneurons are exposed to muscarinic and GABA agonists. On the contrary, when these interneurons are incubated with α1, 5 -HT and histamine receptor agonists the KIR current decreases in amplitude (Borin et al., 2014). The h-current does not appear to be sensitive to 5-HT agonists by its amplitude is strongly reduced when the DA interneurons are exposed to noradrenaline (Pignatelli et al., 2013). Thus, based on the characterization of the h and KIR current it can be hypothesized their multiple and complex regulation can strongly contribute to the plasticity of the OB neuronal network.

Besides serotoninergic and noradrenergic innervation, there is solid evidence that OB is under a massive extrinsic cholinergic innervation, and that the main source of cholinergic afferents is represented by the nucleus of the horizontal limb of the diagonal band (Carson, 1984; Zaborszky et al., 1986).

Experimental observations report the presence of cholinergic innervation from the basal forebrain both in the mitral layer and in the glomerular layer (Ravel et al., 1990; Le Jeune and Jourdan, 1993; Nickell et al., 1994; Kasa et al., 1995; Crespo et al., 1999). The cholinergic terminals preferentially, though not exclusively, innervate the subpopulation of DA-PG interneurons through morphologically symmetric contacts, generally associated with inhibitory synaptic actions (Le Jeune and Jourdan, 1993). However, in contrast to this study, De Saint Jan identified a new subtype of type 2 PG neurons as a target of cholinergic innervation which are neither DA, neither CR nor calbindin immunopositive. These cells express M1 muscarinic receptors and they are characterized by a small cell body, apparent absence of axons and dendrites that innervate a single glomerulus. The activation of these cells by acetylcholine provokes inhibition of OB tufted cells (De Saint Jan, 2021).

Despite these contradictions, the cholinergic system appears to influence many basic functions of OB, including maturation (Halasz and Shepherd, 1983; Kratskin and Belluzzi, 2003), olfactory memory (Ravel et al., 1994; Levy et al., 1995) and odors processing (Nickell and Shipley, 1988; Elaagouby and Gervais, 1992; Linster and Hasselmo, 1997). These findings prompted us to study the effects of this innervation on the activity of dopaminergic neurons in the OB (Pignatelli and Belluzzi, 2008). It was shown that the effect of muscarinic agonists (M_2_ agonist oxotremorine) leads to the reduction of DA periglomerular firing, because of membrane potential hyperpolarization. In this study, we hypothesized that a second messenger leads to the activation of potassium conductance, which is most likely represented by the previously described KIR current.

This muscarinic effect described on DA-PG cells can play a role in the modulation of the signal output from OB to central structures. It can be also relevant to understand the perturbations of cholinergic inputs to the cerebral cortex that occur in Alzheimer's disease.

Glutamatergic projections that make direct synaptic contact on DA-PG cells are innervated by presynaptic terminals (Kosaka et al., 1997) and modulate the release of DA. Indeed, metabotropic glutamate receptors present in DA bulb cells were the subject of electrophysiological studies, which confirmed the presence in this area of two receptor subtypes: mGluR1 and mGluR5 (Martin et al., 1992; Shigemoto et al., 1993; Romano et al., 1995). First, the expression of these receptors was demonstrated by quantitative Real-Time PCR and then, using selective agonists and antagonists of mGluR different subtypes, different electrophysiological parameters were analyzed (Jian et al., 2010). The consequence of this glutamatergic innervation of DA-PG interneurons is an inhibition of neurotransmitter release from olfactory nerve terminals acting on presynaptic D2 receptors (Berkowicz and Trombley, 2000; Ennis et al., 2001; Davila et al., 2003).

Pharmacological activation in bulbar DA cells of mGluR1 receptors produces a depolarization which is presumed to lead to an increase of DA release from the cell, with a consequent reduction in the release of glutamate from the ON axon terminals. On the other hand, mGluR5 was seen to produce the opposite effect. Various hypotheses on the physiological significance of the opposite effects of mGluR1 and mGluR5 on DA can be formulated as suggested by our group (Jian et al., 2010), but in general, this differential regulation mechanism leads to fine regulation of DA excitability.

## Adult Neurogenesis of DA Cells

OB displays another interesting feature, namely, it is one of the two regions of the mammalian CNS that undergo continuous neuronal replacement during adulthood (Gross, 2000). In the subventricular zone (SVZ) of the lateral ventricles, new cells are generated and migrate in the rostral stream toward the OB (Pignatelli and Belluzzi, 2010; Cave and Baker, 2015; Lledo and Valley, 2016; Malvaut and Saghatelyan, 2016). Here they differentiate into diverse subtypes of interneurons that lead to the formation of new granules or periglomerular neurons (Belluzzi et al., 2003; Carleton et al., 2003; Yang, 2008) and finally integrate synaptically into the existing neural network (Belluzzi et al., 2003). The formation of new cells and their integration into the circuitry is essential for maintaining homeostasis in the olfactory bulb.

Interneurons increase in different proportion in the granular and glomerular layer of OB (Kosaka et al., 1987b; McLean and Shipley, 1988; Winner et al., 2002; De Marchis et al., 2007; Batista-Brito et al., 2008). Adult neurogenesis restores the subtypes belonging to the PG population, which express TH, calretinin and a fraction of calbindin glutamatergic interneurons (Lledo and Valley, 2016). In the work by De Marchis et al. (2007) data for adult regeneration of less studied PG subtype cells are also reported. These cells are positive for neurocalcin calcium-binding proteins. Example of BrdU labeled cells co-expressing parvalbumin have also been reported (Whitman and Greer, 2007). The rate of neurogenesis in these three subtypes of PG cells is different and is higher for the PGs expressing calretinin and the lowest in the PGs expressing calbindin indicating that each PG subtype has the unique substitution and/or addition modality (Sakamoto et al., 2014).

Indeed, it was observed that, after 2 months, adult neurogenesis provokes a complete turnover in the granular cell layer, with the cell number remaining stable, while in the glomerular layer the number of new neurons outnumbers about 30% the cell loss (Ninkovic et al., 2007). Intriguingly, the net increase in the number of cells resulting from the added and the lost, in the glomerular layer during adult neurogenesis in the OB, is subtype-specific, as it concerns only two subtypes of interneurons, i.e., calretinin and DA cells (Ninkovic et al., 2007; Adam and Mizrahi, 2011).

The dorsolateral region of SVZ (Fiorelli et al., 2015) is engaged in generating DA progenitor cells characterized by the expression of the transcription factor Pax6 (Merkle et al., 2007; Young et al., 2007; Brill et al., 2008; Fernandez et al., 2011) which is necessary for the development of the DA phenotype (Dellovade et al., 1998; Kohwi et al., 2005; Haba et al., 2009). Other markers for the DA phenotype are the transcription factors Dlx2 and Meis2 (Brill et al., 2008; de Chevigny et al., 2012; Agoston et al., 2014).

It is also important that these newly generated neurons can survive and integrate into the circuitry. The addition of new DA neurons in the olfactory bulb is a plastic, experience-dependent process, and occurs in response to sensory stimuli. Surprisingly, this phenomenon is only specific to DA cells and not to calretinin or calbindin PG cells (Bonzano et al., 2014; Galliano et al., 2021).

Survival and integration processes depend on the abundance of the olfactory input, being strongly enhanced by odor enrichment (Rochefort et al., 2002; Yamaguchi and Mori, 2005; Bonzano et al., 2014, 2016). The DA-PG cells, as commented before, are type 1 cells that make direct contact with the olfactory nerve at the beginning of the bulbar circuitry. Due to this direct connection with the olfactory nerve, DA-PG cells are therefore particularly sensitive to different degrees of olfactory inputs to which they are exposed (Kosaka and Kosaka, 2007). This leads to dynamic control of turnover in a spatial and neuronal subtype-specific manner (Sawada et al., 2011). In fact, in animals that are odor-deprived by either chemical or surgical deafferentation of the OB (Nadi et al., 1981; Kawano and Margolis, 1982; Baker et al., 1983) or naris occlusion (Brunjes et al., 1985) a large reduction in the number of DA cells is observed. This phenomenon can be reverted and affects both pre-existing and adult-generated neurons (Bovetti et al., 2009; Bastien-Dionne et al., 2010). It is not yet clear which mechanisms are involved in these events, but a role is presumed to be played by transcriptional and epigenetic regulators (Banerjee et al., 2013; Bovetti et al., 2013; Bonzano et al., 2016) and microglia (Grier et al., 2016).

## Electrophysiology of DA Neurons During Adult Neurogenesis

Immunohistochemical observations performed in the 80s' showed that mature DA neurons are strictly localized in the glomerular layer (Halasz et al., 1981). However, subsequent observations using a transgenic mice model, indicate that some neurons expressing eGFP under the TH promoter can be also observed in other layers including EPL, MCL and GCL (Baker et al., 2001; Pignatelli et al., 2009; Korshunov et al., 2020a; Kosaka et al., 2020). For these cells, the transcription of the TH gene occurs in absence of significant translational activity (Jeong et al., 2003). For this reason, it has been proposed that these neurons express TH, but dopamine is not produced. Thus, these neurons could be newly neurons observed at different stages of maturation committed to becoming DA neurons (Saino-Saito et al., 2004). We tested this hypothesis with different methods (Pignatelli et al., 2009).

After the characterization of the mature DA cells present in the glomerular layer (Pignatelli et al., 2005, 2012), a comparative analysis of the cells presents in the other layers that are assumed to be immature was carried out (Pignatelli et al., 2009). Using TH-GFP transgenic mice (Sawamoto et al., 2001) in which the eGFP gene is located under the TH promoter, we were able to observe that TH^+^ neurons present in the mitral and EPL layers have an eGFP fluorescence lower than that present in the cells of the glomerular layer, but still sufficient to allow the identification of these cells.

TH-GFP neurons recorded in EPL are autorhythmic cells, as they show a spontaneous firing almost indistinguishable from that of the mature DA neurons present in the GL. Autorhythmicity in these cells is due to the presence of the same voltage-dependent currents: Na^+^ persistent (INa_P_) and a T-type Ca^2+^; as it occurs in the spontaneous activity of mature DA neurons (Pignatelli et al., 2009, 2012). Autorhythmicity instead is not observed in TH-GFP cells present in the mitral layer, where the T-type calcium current, which is one of the currents involved in the pacemaker mechanism, is absent. However, these cells can generate trains of action potentials in response to depolarizing pulses.

High intracellular Cl^−^ concentration is a marker for immature neurons (Ben-Ari et al., 2007). To demonstrate the hypothesis that the TH-GFP cells present in the three layers of the olfactory bulb (mitral, EPL and GL) are cells with an increasing degree of maturity, the intracellular concentration of Cl^−^ was measured (Pignatelli et al., 2009). The results showed that neurons in the EPL and mitral layer possess a higher intracellular Cl^−^ concentration than those within the GL, thus confirming this hypothesis.

One further observation supporting the hypothesis of maturation of glomerular DA neurons is based on experiments of TH-GFP cells stimulation from the olfactory nerve. Indeed, to complete their maturation they must establish an asymmetric connection with fibers derived from the olfactory epithelium (Brunjes et al., 1985; Stone et al., 1991; Wilson and Wood, 1992; Cho et al., 1996; Toida et al., 2000).

Our electrophysiological analysis showed that most (75%) of the EPL cells respond upon ON stimulation with a monosynaptic EPSP and that this response can be reverted by incubation with kynurenate (Pignatelli et al., 2009). On the other hand, the cells present in the mitral layer, which were supposed to be more immature, do not respond synaptically to ON stimulation. However, mitral TH-GFP neurons respond to the focal application of glutamate, which is the same neurotransmitter released by the ON on mature DA cells (Pignatelli et al., 2009). This suggests that these cells already have functional receptors for glutamate, but not a synaptic connection with the olfactory nerve.

All these observations suggest that the weakly fluorescent TH-GFP cells observed in the mitral layer represent newly generated DA cells that arrived in the OB which stopped their migration process at this level. To reach their destination in the glomerular layer, these immature cells require a consensus signal coming from the glomerular region and the passage across the EPL that brings to the end their differentiation toward the DA phenotype. The signal is still unknown, but previously data from our group suggests that the establishment of synaptic contacts with the olfactory nerve could be the trigger (Pignatelli et al., 2009).

## CR-PG Cells in the Olfactory Bulb

Calretinin is a calcium-binding protein commonly used as a marker for several cellular populations in the nervous system (Jacobowitz and Winsky, 1991; Miettinen et al., 1992; Resibois and Rogers, 1992; Huberman et al., 2008; Barinka and Druga, 2010). Under this aspect, the olfactory bulb represents no exception, considering that most of the neurons in different OB layers are positive to CR staining. In the glomerular layer alone, studies have calculated that CR cells account for a percentage of all the juxtaglomerular cells ranging from 28 to 44% (Kosaka and Kosaka, 2007; Panzanelli et al., 2007; Parrish-Aungst et al., 2007; Whitman and Greer, 2007) representing the most abundant PG interneurons in the glomerular layer of the olfactory bulb. CR-PG cells are also the most abundant PG cells generated postnatally. These neurons are mostly generated after birth, in adulthood, from neural stem cells derived from the dorsal septal and subventricular areas and have homogeneous morphological and electrophysiological properties (Benito et al., 2018).

From the temporal point of view, the production of new CR-PG cells peaks around birth and continues throughout life. This differs from the other subtypes of PG cells (dopaminergic and calbindin immunoreactive) whose production is maximum during embryogenesis and decreases after birth (De Marchis et al., 2007; Ninkovic et al., 2007; Batista-Brito et al., 2008; Li et al., 2011; Weinandy et al., 2011).

A recent work by the De Saint Jan group showed that the newly generated CR-PG cells can be distinguished from the other generated neurons because they seem to maintain properties of immaturity. Other subtypes of neurons generated after birth are rapidly integrated into the pre-existing circuitry of olfactory bulbs, while these cells are not inserted into the activity of the local network, as if they never fully mature. The function of this reserve of immature cells in this bulbar area is still unclear (Benito et al., 2018).

The presence of calretinin-positive neurons has been described also in the accessory olfactory bulb of the mouse, where inhibitory interneurons play a role related to behavior following the exposition to social odors (Jacobowitz and Winsky, 1991; Maksimova et al., 2019).

CR neurons, together with calbindin-positive neurons, constitute the type 2 PG cells identified by the Kosaka group (Kosaka, 2007; Kosaka and Kosaka, 2009b). As previously described, the processes of these PG cells create contact with other classes of OB neurons but do not receive direct synaptic connections from the olfactory nerve axons.

From the morphological point of view, CR-PG cells are described as axonless neurons characterized by compact round-shaped soma with dendrites. When compared to the other JG cells, their soma size results to be the smallest, with an average diameter varying from 6 to 8 μm (Batista-Brito et al., 2008; Fogli Iseppe et al., 2016). In addition, differently from the other JG neurons, the soma diameters of CR cells are unimodally and normally distributed, suggesting the existence of a quite homogeneous population.

Consistent with this classification, has recently been confirmed that CR-PG cells do not receive synaptic inputs from OSNs (Najac et al., 2011). They are not innervated by olfactory sensory neurons and receive few synaptic inputs from mitral or tufted cells at excitatory synapses where NMDA receptors predominate (Benito et al., 2018) but establish GABAergic type dendrodendritic synapses with mitral and tufted cells (Panzanelli et al., 2007).

In adult animals, CR-PG interneurons are considered to participate in the inhibitory glomerular circuitry via GABA release (Burton, 2017). In the murine olfactory bulb, the majority of CR-PG cells express indeed GABAergic markers, as shown both in knock-in animals (Panzanelli et al., 2007) and by immunohistochemistry (Kosaka, 2007; Parrish-Aungst et al., 2007). In addition, type 2 CR-PG cells have been associated with cholinergic immunoreactivity. Notably, CR staining does not colocalize with cholinergic cells, which characterizes instead the large majority of CB-positive PG neurons (Krosnowski et al., 2012). On the other side, CR cells in the glomerular region show a nontrivial expression of nitric acid synthase, while this biomarker is rarely associated with the calbindin-positive population of periglomerular cells (Kosaka and Kosaka, 2007).

## CR-PG Cells: Electrophysiological Profile

From the electrophysiological point of view, CR-PG neurons display several peculiar characteristics. First, CR cells in the glomerular region possess small membrane capacitance and elevated input resistance, calculated to be approximately 4 pF and 2 GΩ, respectively. Notably, the variance of these measurements across cells is low, in agreement with the idea of a unique neuronal population previously introduced. When CR-PG neurons are recorded with voltage-clamp protocols, the two mains ionic conductances that can be distinguished are a transient outward A-type K^+^ and fast transient Na^+^ current (Fogli Iseppe et al., 2016; Benito et al., 2018). It is interesting to notice that being both these two currents only transiently active, the electrical behaviour of the CR-PG cells become purely ohmic under prolonged depolarizing stimulation. An additional consequence, which clearly distinguishes the electrophysiological characteristics of this group of periglomerular cells from the other classes, can be observed in current-clamp conditions. When a depolarization is applied, the CR neurons can generate only one complete action potential, as opposed to the train of spikes that dopaminergic neurons can generate (Figures 2A,B) and remain silent for the remaining duration of the stimulus; this observation was also confirmed by De Saint Jan group (Benito et al., 2018).

However, the two transiently activated currents do not constitute the entire set of conductances that this group of periglomerular neurons expresses. Carefully dissecting the remaining current observable during long depolarizing steps in voltage clamp, it is indeed possible to pharmacologically isolate a small persistent L-type Ca^2+^ current (Fogli Iseppe et al., 2016). Lastly, CR-PG cells possess an h-current, as clearly indicated also by the time-dependent depolarizing sag produced in response to the injection of a hyperpolarizing current.

## CR-PG Cells: Possible Role in Bulbar Circuitry

Some studies have investigated the role of the CR-PG cells in the complex process of olfactory information processing (Fogli Iseppe et al., 2016; Benito et al., 2018; Sanz Diez et al., 2019). Despite a comprehensive understanding of the synaptic connectivity inside the glomerulus is still missing (Vaaga and Westbrook, 2016), recent findings can help in defining some hypotheses. Differently from dopaminergic PG cells, CR neurons are anaxonic (Kosaka and Kosaka, 2010). Thus, their action is confined to the intraglomerular region, where they could regulate their activity by self-inhibition or self-modulation processes (Gire and Schoppa, 2009; Kosaka and Kosaka, 2011).

In addition, CR cells have been shown to have weak connectivity with the rest of the glomerular network (Benito et al., 2018; Sanz Diez et al., 2019). Coherently with the histological investigations, these neurons show few EPSCs in response to ON stimulation. The same is generally true for inhibitory postsynaptic currents, which suggests that inputs received from interneurons nearby are rare as well. Conversely, the stimulation of centrifugal fibers originated in the basal forebrain has been described to elicit GABAergic responses in the CR-PG neurons (Sanz Diez et al., 2019).

Taken all together, the findings described above support the idea of an extremely focused role for the CR-PG population. In particular, considering their extremely peculiar electrophysiological profile, these neurons have been proposed to improve the signal-to-noise ratio, acting as a filter against randomly occurring ON-evoked excitatory stimuli (Fogli Iseppe et al., 2016).

What is the function of this neuronal population, which is very abundant and continuously renewed, is still difficult to identify. The work of Benito et al. proposes an additional hypothesis based on their evidence that indicates these CR-positive cells as immature. These cells could therefore constitute a reserve pool of latent and not completely differentiated interneurons that could be recruited on request. The authors also hypothesize that these cells can mature because of sensory input but also convert into another cellular subtype of PG. This phenomenon would lead to greater recruitment of PG cells generated in the adult (Benito et al., 2018).

## Concluding Remarks

In the CNS, interneurons play a fundamental role in modulating the transmission of nerve information. Depending on the brain areas and the different neuronal functions, interneurons develop in subtypes with different morphologically, molecularly, and electrophysiological properties.

The study of interneurons is important for at least two reasons: on one hand, it allows us to understand complex brain circuits (Maccaferri and Lacaille, 2003). On the other hand, it provides more information on neurodevelopmental disorders that are associated with interneuron malfunction (Fang et al., 2014), such as autism spectrum disorder and Tourette's syndrome (Ashwin et al., 2014).

The mammalian OB is a part of the brain where it is possible to identify a large percentage of diversified interneurons that are continuously generated in postnatal and adult periods (Batista-Brito and Fishell, 2009; Bartolini et al., 2013; Kepecs and Fishell, 2014).

The olfactory bulb interneurons have another peculiar characteristic: it has been observed that the proportion of inhibitory interneurons concerning excitatory neurons is extremely higher (100: 1 ratio, compared to other brain regions at a 1:5 ratio (Kim et al., 2020).

In this review, we focus on the description of dopaminergic and calretininergic cells, two populations of interneurons that play a role in olfactory information processing.

DA cells present in the olfactory bulb are the largest population of dopaminergic neurons in the brain and have been the object of many studies describing both the morphology and the electrophysiological properties of these cells. The functional role of these interneurons is still unclear, especially when we look at the DA cells that are generated in this area in adulthood.

These interneurons are supposed to play important roles in the control of glomerular output information in the OB (Banerjee et al., 2015; Liu et al., 2016; Burton, 2017; Pignatelli and Belluzzi, 2017; Vaaga et al., 2017; Shao et al., 2019). To understand how DA neurons contribute to signal processing, it is important to know their connections. A recent work (Kosaka et al., 2020) has shown a great heterogeneity of the DA cells in the olfactory bulb, and that still little is known about the synaptic connections of each cell type described and their role in the bulbar circuitry.

However, electrophysiological analyses of DA olfactory bulb neurons gave the possibility to understand important aspects of the voltage-conductance involved in the excitability profile and synaptic modulation of these cells (Pignatelli et al., 2005, 2013; Pignatelli and Belluzzi, 2008; Jian et al., 2010; Borin et al., 2014).

To answer the question of why DA neurons are continuously produced in the olfactory bulb, we need to consider the characteristics of the olfactory structure and how the adult neurogenesis process occurs. In mice, adult neurogenesis is a process in which new neurons are added and odor memory is improved. This cell increase is stimulated by enriched odor exposure, even if constrained to keep the number of neurons constant in time (Rochefort et al., 2002). This means that the new neurons are not added but replace the existing ones. The reason for this process could be that the olfactory bulb needs to adjust the circuitry following new olfactory experiences.

The data reported in this review can give a possible explanation of how this process occurs. DA cells are continuously generated over time and migrate in the OB until they reach the mitral cell layer. Here, they remain in a standby condition waiting to receive a consensus signal to finish their differentiation process and reaching the final position in the glomerular layer (Pignatelli et al., 2009). Then, we hypothesize that the differentiation and maturation will be completed when immature cells in the mitral layer will start making active synapses within the glomerular layer. However, many cells will not follow this path and will therefore undergo to apoptosis and death, as indicated by evidence that shows most of the newly generated cells arriving from the SVZ being eliminated before reaching the olfactory bulb.

All these data, together with the recent observation that DA cells undergo to adult neurogenesis also in humans (Inta et al., 2015) and their capacity to integrate into neuronal circuits make them an attractive tool for replacement strategies in cases of loss of dopaminergic neurons, such as Parkinson's disease (Lledo and Saghatelyan, 2005).

Another interesting aspect concerning DA cells was recently discussed in a review (Korshunov et al., 2020b), which describes how the two sensory systems, the retina and OB, are remarkably similar. Both areas have an anatomical structure made up of layers where sensory signals follow a precise sequence, and both areas utilize lateral inhibition. Moreover, the presence of interneurons that release DA is important to have a correct interpretation of both olfactory and visual signals.

We described that CR interneurons located in the glomerular layer are the most abundant neuronal population in the olfactory bulb. As in the case of DA cells, CR-PG cells are continuously produced in adulthood, but surprisingly in some cases, they do not integrate into pre-existing olfactory bulb networks for a long time (Benito et al., 2018).

Electrophysiological studies also reveal another unusual feature of these cells. CR-PG cells when stimulated by a train of excitatory inputs, sufficiently close to depolarize stably the membrane, respond with a single action potential (Fogli Iseppe et al., 2016). This behaviour is due to the inactivation of the two main voltage-dependent currents expressed by these cells, an A-type K^+^ and a classic Na^+^ current. Therefore, if a train of excitatory inputs arrives, the cell will fire only one complete spike before becoming completely unexcitable.

What could be the role of these cells in the olfactory bulb? They are inhibitory interneurons and are positioned at the entry of the bulbar network. It has been proposed that they could improve the signal-to-noise ratio of the network. If a random excitatory input, representing a meaningless signal (noise), arrives, CR-PG cells will activate and lead to an inhibition of the projection neurons (Fogli Iseppe et al., 2016).

In conclusion, the information presented in this review gave some initial clues on the functions of these two populations of interneurons, but further studies are needed to completely define their role in the olfactory sensory process.

## Author Contributions

SC, AP, and AFI conceived the review and wrote the manuscript. SC, AP, and FC checked and revised the manuscript. All authors contributed to the article and approved the submitted version.

## Funding

This research was supported by the Italian Ministry of University and Research (PRIN 2019-PRA.A-PA_001) and by local funds of the University of Ferrara (2020-FAR.L-PA_001).

## Conflict of Interest

The authors declare that the research was conducted in the absence of any commercial or financial relationships that could be construed as a potential conflict of interest.

## Publisher's Note

All claims expressed in this article are solely those of the authors and do not necessarily represent those of their affiliated organizations, or those of the publisher, the editors and the reviewers. Any product that may be evaluated in this article, or claim that may be made by its manufacturer, is not guaranteed or endorsed by the publisher.

## Abbreviations

CB: calbindin-28K
CR: calretinin
DA: dopaminergic
eGFP: enhanced green fluorescent protein
EPL: external plexiform layer
EPSCs: excitatory postsynaptic currents
ET: external tufted
GABA: γ-aminobutyric acid
GAD: acid decarboxylase
Gcs: granule cells
GL: glomerular layer
ICa_T_: T-type Ca^2+^ current
INa_P_: Na^+^ persistent current
JGcs: juxtaglomerular cells
KIR: inward rectifier current of potassium
OB: olfactory bulb
OSNs: olfactory sensory neurons
PG: periglomerular
sSA: short axon surface
SVZ, subventricular zone, TH: tyrosine hydroxylase.
